# Oncogenetic landscape and clinical impact of *IDH1* and *IDH2* mutations in T-ALL

**DOI:** 10.1186/s13045-021-01068-4

**Published:** 2021-05-03

**Authors:** Mathieu Simonin, Aline Schmidt, Christophe Bontoux, Marie-Émilie Dourthe, Etienne Lengliné, Guillaume P. Andrieu, Ludovic Lhermitte, Carlos Graux, Nathalie Grardel, Jean-Michel Cayuela, Françoise Huguet, Isabelle Arnoux, Stéphane Ducassou, Elizabeth Macintyre, Virginie Gandemer, Hervé Dombret, Arnaud Petit, Norbert Ifrah, André Baruchel, Nicolas Boissel, Vahid Asnafi

**Affiliations:** 1 Laboratory of Onco-Hematology, Assistance Publique-Hôpitaux de Paris (AP-HP), Hôpital Necker Enfants-Malades, Université de Paris, 149 rue de Sèvres, 75015 Paris, France; 2 Department of Pediatric Hematology and Oncology, Assistance Publique-Hôpitaux de Paris (AP-HP), Armand Trousseau Hospital, Sorbonne Université, Paris, France; 3 Institut Necker-Enfants Malades (INEM), Institut National de la Santé et de la Recherche Médicale (Inserm) U1151, Paris, France; 4 PRES LUNAM, CHU Angers service des Maladies du Sang, INSERM U 892, Angers, France; 5 Department of Pediatric Hematology and Immunology, Assistance Publique-Hôpitaux de Paris (AP-HP), Robert Debré Hospital, University Paris Diderot, Paris, France; 6 Université Paris Diderot, Institut Universitaire d’Hématologie, EA-3518, Assistance Publique-Hôpitaux de Paris, University Hospital Saint-Louis, Paris, France; 7 Department of Hematology, Université Catholique de Louvain, CHU UCL Namur - site Godinne, Yvoir, Belgium; 8 Laboratory of Hematology, CHRU - Inserm U1172, Lille, France; 9 Inserm U1172, Lille Cedex, France; 10 Laboratory of Hematology, Saint-Louis Hospital, AP-HP, Paris, France; 11 Department of Hematology, CHRU - Institut Universitaire de Cancer Toulouse - Oncopole, Toulouse, France; 12 Laboratory of Hematology, Marseille University Hospital Timone, Marseille, France; 13 Pediatric Hematology-Oncology Department, Centre Hospitalier Universitaire (CHU), Bordeaux, France; 14 Department of Pediatric Hematology and Oncology, University Hospital of Rennes, Rennes, France

**Keywords:** IDH1, IDH2, T-ALL

## Abstract

*IDH1* and *IDH2* mutations *(IDH1/2*^*Mut*^) are recognized as recurrent genetic alterations in acute myeloid leukemia (AML) and associated with both clinical impact and therapeutic opportunity due to the recent development of specific *IDH1/2*^*Mut*^ inhibitors. In T-cell acute lymphoblastic leukemia (T-ALL), their incidence and prognostic implications remain poorly reported. Our targeted next-generation sequencing approach allowed comprehensive assessment of genotype across the entire *IDH1* and *IDH2* locus in 1085 consecutive unselected and newly diagnosed patients with T-ALL and identified 4% of, virtually exclusive (47 of 49 patients), *IDH1/2*^Mut^. Mutational patterns of *IDH1/2*^Mut^ in T-ALL present some specific features compared to AML. Whereas *IDH2*^R140Q^ mutation was frequent in T-ALL (25 of 51 mutations), the *IDH2*^R172^ AML hotspot was absent. *IDH2* mutations were associated with older age, an immature phenotype, more frequent *RAS* gain-of-function mutations and epigenetic regulator loss-of-function alterations (*DNMT3A* and *TET2*). *IDH2* mutations, contrary to *IDH1* mutations, appeared to be an independent prognostic factor in multivariate analysis with the *NOTCH1/FBXW7/RAS/PTEN* classifier. *IDH2*^Mut^ were significantly associated with a high cumulative incidence of relapse and very dismal outcome, suggesting that *IDH2*-mutated T-ALL cases should be identified at diagnosis in order to benefit from therapeutic intensification and/or specific *IDH2* inhibitors.

**Supplementary Information:**

The online version contains supplementary material available at 10.1186/s13045-021-01068-4.

## Introduction

T-cell acute lymphoblastic leukemia (T-ALL) is aggressive neoplasms resulting from the proliferation of T-lymphoid progenitors blocked at thymic stages of differentiation and account for 15% and 25% of pediatric and adult ALLs, respectively [1]. T-ALL is associated with a wide range of acquired genetic abnormalities that contribute to developmental arrest and abnormal proliferation [2]. Although intensive treatment protocols have markedly improved the outcomes of children with T-ALL, cure rates remain below 60% for adults and 85% for children [3–5]. The prognosis is particularly poor in relapsing patients, justifying the development of novel targeted therapies [6, 7]. For example, alterations affecting epigenetic factors may offer novel targeted therapeutic approaches in high-risk T-ALL [8].

Whole-genome sequencing of AML identified acquired mutations in isocitrate dehydrogenase 1 and 2 (*IDH1/2)* [9]. These paralogous genes encode two enzymes with distinct localizations (cytoplasmic for IDH1 and mitochondrial for IDH2). Both catabolize the conversion of isocitrate to α-ketoglutarate (α-KG). Gain-of-function *IDH1/2* mutations (*IDH1/2*^*Mut*^) confer a neomorphic activity on the encoded enzymes, leading to the conversion of α-KG to 2-hydroglutatarate (2-HG) in a NAD phosphate-dependent manner [10]. Accumulation of the oncometabolite 2-HG induces multiple cellular alterations, including chromatin methylation and cellular differentiation, by inhibiting α-KG-dependent enzymes related to DNA methylation, such as Tet oncogene family members (TET2, TET3) [11]. *IDH1/2*^Mut^ have been reported in 10 to 20% of AML cases, when they are predominantly located in the active site of the enzyme (*IDH1*^R132^, *IDH2*^R140Q^ and *IDH2*^R172^). *IDH1/2*^*Mut*^ in AML are associated with prognostic impact influenced by the genetic context [12, 13]. Importantly, specific drugs targeting mutant *IDH1* or *IDH2* have recently shown promise in *IDH1/2*^*Mut*^ refractory or relapsed AML patients [14, 15].

In T-ALL, *IDH1/2*^*Mut*^ have been partially explored and their prognostic impact poorly reported [16, 17]. We now provide the first comprehensive analysis and oncogenetic landscape of *IDH1/2*^Mut^ in a cohort of 1085 T-ALL patients, when the nearly 4% of *IDH1/2*^*Mut*^ are associated with extremely poor prognosis, specifically in *IDH2*-mutated cases.

## Methods

### Patient’s protocol and clinical trials

Diagnostic peripheral blood or bone marrow samples from 1085 adults and children with T-ALL were analyzed after informed consent was obtained at diagnosis according to the Declaration of Helsinki. Among the 1085 T-ALL analyzed, 215 adult patients aged from 16–59 years were included in the GRAALL03/05 trials (details provide in supplementary) which were registered at clinicaltrials.gov (GRAALL-2003, #NCT00222027; GRAALL-2005, #NCT00327678). and 261 pediatric patients aged from 1 to 19 years were treated in 10 French pediatric hematology departments, members of the FRALLE study group, according to the FRALLE 2000 T guidelines (Additional file 2: Fig. S5 and Additional file 1: Table S3).

### Gene mutation screening

A custom capture Nextera XT gene panel (Illumina, San Diego, CA) targeting all coding exons and their adjacent splice junctions of 80 genes was designed, based on available evidence in hematological neoplasms (Additional file 1: Table S1). DNA Libraries were prepared using Nextera Rapid Capture Enrichment protocol and underwent 2 × 150 bp paired-end sequencing on Illumina MiSeq sequencing system with MiSeq Reagent Kit v2 (Illumina). Briefly, sequence reads were filtered and mapped to the human genome (GRCh37/hg19) using in-house software (Polyweb, Institut Imagine, Paris). Annotated variants were selected after filtering out calls according to the following criteria: (1) coverage < 30×, < 10 alternative reads or variant allelic fraction (VAF) < 7%; (2) polymorphisms described in dbSNP, 1000Genomes, EVS, Gnomad and EXAC with a calculated mean population frequency > 0.1%. Non-filtered variants were annotated using somatic database COSMIC (version 78) and ProteinPaint (St Jude Children’s Research Hospital – Pediatric Cancer data portal). Lollipop plots were generated with ProteinPaint (https://pecan.stjude.org/#/proteinpaint).

### Immunophenotypic and molecular characterization of T-ALL samples

Peripheral blood or bone marrow T-ALL samples were analyzed for immunophenotype, fusion transcripts (SIL-TAL1, CALM-AF10), oncogenic transcripts (HOXA9, TLX1 and TLX3) and T-cell receptor (TCR) recombination and *NOTCH1/FBXW7/RAS/PTEN* mutations, as previously described [4, 18, 19].

### Minimal residual disease assessment

Immunoglobulin/T-cell receptor (Ig/TCR) gene rearrangement-based Minimal Residual Disease (MRD) evaluation was centrally assessed for patients who reached complete remission after the first induction cycle, on BM samples after induction (MRD1). MRD was centrally assessed by real-time quantitative allele-specific oligonucleotide PCR and interpreted according to EuroMRD group guidelines [20–22].

### Statistical analysis

Comparisons for categorical and continues variables between *IDH1*^*Mut*^* or IDH2*^*Mut*^ and *IDH*^*WT*^ subgroups were performed with Fisher's exact test and Mann–Whitney test, respectively. Overall survival (OS) was calculated from the date of diagnosis to the last follow-up date censoring patients alive. The cumulative incidence of relapse (CIR) was calculated from the complete remission date to the date of relapse censoring patients alive without relapse at the last follow-up date. Relapse and death in complete remission were considered as competitive events. Univariate and multivariate analyses assessing the impact of categorical and continuous variables were performed with a Cox model. Proportional-hazards assumption was checked before conducting multivariate analyses. In univariate and multivariate analyses, age and log10(WBC) were considered as continuous variables. All analyses were stratified on the trial. Variables with a *p* value less than 0.1 in univariate analysis were included in the multivariable models. Statistical analyses were performed with STATA software (STATA 12.0 Corporation, College Station, TX). All p-values were two-sided, with *p* < 0.05 denoting statistical significance. Circos plots were generated using R software.

## Results and discussion

### Incidence of *IDH1* and* IDH2* mutations in 1085 T-ALL

**Fig. 1:**
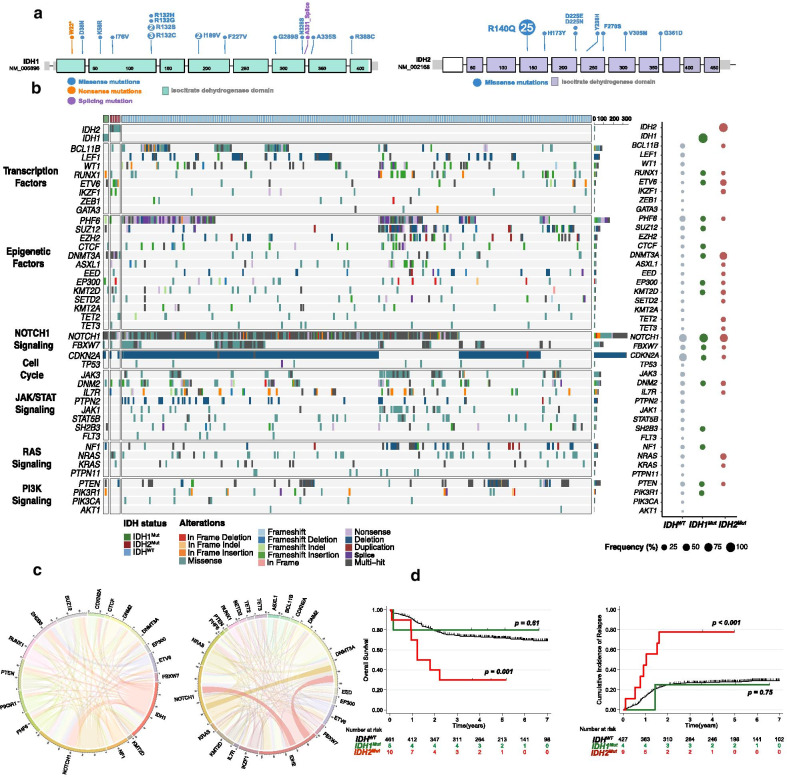
*IDH1* and *IDH2* mutations in the GRAALL03/05 and FRALLE2000 studies. **a** Lollipop plots indicating the observed mutations for each *IDH* gene and their consequences. **b** Oncoplot depicting the genetic anomalies observed in *IDH1/2-*Mutated or Wild type T-ALL cases of the GRAALL03/05 and FRALLE2000 studies. Genes are classified by functional groups. The right panel indicates the overall frequency of alterations per gene. **c** The circos plots depict the co-occurrences in genetic lesions observed in *IDH1* (left panel) and *IDH2* mutated T-ALL (right panel). **d** Clinical impact of *IDH1* and *IDH2* mutations in the GRAALL0305 and FRALLE2000 studies. Overall survival (left panel) and cumulative incidence of relapse (right panel). The red curve represents the *IDH2*-mutated patients, the green curve the *IDH1*-mutated patients and the black curve the *IDH*^*Wt*^ patients

A total of 51 (4%) mutations, mainly clonal, in either *IDH1* or *IDH2* were apparent in 49 cases (Fig. 1a and Additional file 1: Table S2, Additional file 2: Figs. S2, S3). *IDH1* mutations were identified in 19 T-ALL cases (2%) and *IDH2* mutations in 32 cases (3%). *IDH1/2*^*Mut*^ were mutually exclusive except in 2 cases. The *IDH2*^R140Q^ mutation was the most prevalent mutation affecting *IDH2* (*n* = 25, 78%). We identified 7 *IDH1* mutations located in the R132 hotspot (37% of *IDH1* mutations), 3 cases with *IDH1*^R132C^ mutation, 2 with *IDH1*^R132S^, 1 with *IDH1*^R132H^ and *IDH1*^R132G^ mutation. The most common *IDH2* mutations in AML occur at R140 followed by residue *IDH2*^*R*172^. The latter mutation is virtually the only *IDH* mutation found in angio-immunoblastic T cell lymphoma, reported in about 30% of cases (Additional file 2: Fig. S1) [23]. *IDH2*^*R*172^ mutation has also been rarely and inconsistently described in peripheral T-cell lymphoma not otherwise specified (NOS) with *T*-follicular helper (*T*_FH_) phenotype [24, 25]. In striking contrast, *IDH2*^*R*172^ was not reported in our series of T-ALL. *IDH1*^R132^, the most frequent *IDH1* mutation reported in our cohort, has recently been recognized to cooperate with NOTCH1 activation in a T-ALL mouse model [26]. These results highlight the specific consequence associated with *IDH1/2*^*Mut*^ subtype during immature T-cell development.

### Clinico-biological characteristics of ***IDH1/2***^Mut^ in GRAALL and FRALLE-treated T-ALLs

We then investigated the clinical characteristics linked to *IDH1/2*^Mut^ in a subset of 476 patients, including 215 adults enrolled in the GRAALL-2003/2005 trials and 261 children enrolled in the FRALLE-2000 trial (Table 1 and Supplemental Methods). The incidence of *IDH1/2*^Mut^ in this cohort was 3% (15/476). *IDH1* mutations were detected in 5 patients (4 adult and 1 pediatric case), and *IDH2* mutations were identified in 10 (6 adult and 4 pediatric cases) (Additional file 2: Fig. S2). *IDH2*R^140Q^ was the most frequent mutation (*n* = 7, 70%) and was most prevalent in adults' patients (*n* = 6/7, 86%). Overall, *IDH1/2*^Mut^ were observed in 5% of adults and 2% of children (*p* = 0.1).

**Table 1:** Clinico-biological and outcome characteristics of adult and pediatric T-ALL (GRAALL and FRALLE protocols) according to *IDH1/2* status

Variable	*IDH2*^*Mut*^ (*n* = 10)	*p* value^2^	Overall (*n* = 476)	*p* value^2^	*IDH1*^*Mut*^ (*n* = 5)
Male	7/10 (70%)	0.72	357/476 (75%)	0.34	5/5 (100%)
Age (y)^1^	47.6 (3.6–59.1)	**0.01**	15.3 (1.1–59.1)	0.26	21.6 (5.4–56.5)
WBC (G/L)^1^	9 (1–400)	**0.01**	64 (0–980)	0.60	80 (4–110)
CNS involvement	1/10 (10%)	0.99	51/474 (11%)	0.99	0/5 (0%)
*Immunophenotype*					
ETP phenotype	3/5 (60%)	**0.04**	56/307 (18%)	0.54	1/4 (25%)
Immature (IM0/δ/γ)	5/7 (71%)	**0.006**	89/419 (21%)	0.99	1/5 (20%)
Cortical (IMB, preαβ)	0/7 (0%)	**0.007**	211/419 (50%)	0.68	2/5 (40%)
Mature TCRαβ	1/7 (14%)	0.99	66/419 (16%)	0.99	0/5 (0%)
Mature TCRγδ	1/7 (14%)	0.99	53/419 (13%)	0.12	2/5 (40%)
*Oncogenetic classification*					
*TLX1*	0/8 (0%)	0.60	54/415 (13%)	0.99	0/5 (0%)
*TLX3*	1/8 (12%)	0.99	72/415 (17%)	0.21	2/5 (40%)
*SIL-TAL1*	0/8 (0%)	0.61	57/415 (14%)	0.99	0/5 (0%)
*CALM-AF10*	0/8 (0%)	0.99	13/415 (3%)	0.99	0/5 (0%)
High-risk classifier	8/10 (80%)	**0.03**	209/476 (44%)	0.99	2/5 (40%)
*Treatment response*					
Rapid prednisone response	3/10 (30%)	0.12	259/467 (55%)	0.66	2/5 (40%)
Complete Remission	9/10 (90%)	0.54	440/476 (92%)	0.32	4/5 (80%)
MRD1 > 10^–4^	1/1 (100%)	0.36	123/340 (36%)	0.99	1/4 (25%)
Allo-HSCT	2/10 (20%)	0.99	101/456 (22%)	0.99	1/5 (20%)
*Outcome*					
4-year CIR (95% CI)	78% (49;97)	** < 0.001**^**3**^	29% (25;33)	0.75^3^	25% (4;87)
4-year OS (95% CI)	30% (7;58)	**0.001**^**3**^	71% (67;75)	0.61^3^	80% (20;97)

Univariate and multivariate analysis^3^						
	Univariate			Multivariate		
CIR	SHR	95%CI	p	SHR	95%CI	p
Age	1.01	(0.98; 1.03)	0.57	-	-	-
CNS	1.57	(0.85; 2.59)	0.08	1.33	(0.80; 2.20)	0.28
Log(WBC)	1.62	(1.2; 2.18)	**0.002**	1.63	(1.20; 2.22)	**0.002**
Prednisone response	0.67	(0.47; 0.95)	**0.03**	1.00	(0.68; 1.46)	0.99
High-risk Classifier	2.78	(1.94; 3.99)	** < 0.001**	2.62	(1.81; 3.79)	** < 0.001**
*IDH2*^*Mut*^	4.28	(1.99; 9.23)	** < 0.001**	4.06	(1.84; 8.96)	**0.001**
**OS**	HR	95%CI	p	HR	95%CI	p
Age	1.03	(1.01; 1.05)	**0.001**	1.04	(1.02; 1.07)	** < 0.001**
CNS	2.00	(1.28; 3.14)	**0.002**	1.67	(1.02; 1.07)	**0.03**
Log(WBC)	1.99	(1.48; 2.67)	** < 0.001**	2.00	(1.46; 2.76)	** < 0.001**
Prednisone response	0.54	(0.38; 0.76)	** < 0.001**	0.85	(0.59; 1.24)	0.41
High-risk Classifier	2.93	(2.06; 4.17)	** < 0.001**	2.90	(2.00; 4.19)	** < 0.001**
*IDH2*^*Mut*^	3.56	(1.66; 7.65)	**0.001**	1.98	(0.86; 4.57)	0.11

*p*-values < 0.05 are indicated in bold

MRD1 correspond to MRD evaluation after induction and was performed by allele-specific oligonucleotides polymerase chain reaction. T-cell receptor status and oncogenic were performed as described in supplemental methods. *IDH1*^*Mu*t^ and *IDH2*^*Mut*^ were statistically compared to *IDH1*^*WT*^ and *IDH2*^*WT*^ patients, respectively

T-ALL: T-cell acute lymphoblastic leukemia; WBC, white blood count; CNS, central nervous system; ETP, early thymic precursor; *High Risk* classifier, *NOTCH1/FBXW7-RAS/PTEN* classifier as previously described [3, 4]; CR, complete remission; MRD, minimal residual disease; Allo-HSCT, allogenic hematopoietic stem cell transplantation; CIR, cumulative incidence of relapse; OS, overall survival; HR: hazard ratio, SHR: specific hazard ratio, CI: confidence interval

^1^Statistics presented: Median (Minimum–Maximum)

^2^Statistical tests performed: Fisher's exact test; Wilcoxon rank-sum test

^3^Univariate and multivariate Cox analyses stratified on protocol

### *IDH1 and IDH2* mutations are associated with both specific clinical and mutational profiles

Patients with *IDH2*^Mut^ were significantly older than *IDH*^*WT*^ (median 47.6 years vs 15.0, *p* = 0.01). *IDH2*^*Mut*^ were associated with an immature immunophenotype (5/7, 71% vs 83/407, 20%, *p* = 0.006) and ETP-phenotype (3/5, 60% vs 52/298, 17%, *p* = 0.04). In line with this, *IDH2*^Mut^ correlated positively with abnormalities known to be associated with an immature phenotype, including *RAS* (50% vs 11%, *p* = 0.02), *ETV6* (40% vs 3%, *p* < 0.01), *DNMT3A* (70% vs 3%, *p* < 0.01), *IKZF1* (20% vs 2%, *p* = 0.02) and *TET2* (20% vs 2%, *p* = 0.04) mutations (Fig. 1b, c). *IDH2*^Mut^ were mutually exclusive with *SIL-TAL1* + cases, associated with a mature TCRαβ lineage. Interestingly, contrary to *IDH2*-mutated cases, *IDH1*^*Mu*t^ did not statistically differ from *IDH*^*WT*^ patient regarding age, immunophenotype or mutational co-occurrence.

### *IDH2 *mutations, but not *IDH1*, are associated with a poor prognosis in T-ALL

To investigate the prognostic value of *IDH1/2*^*Mut*^, survival analyses were performed on the 476 patient cohort. *IDH1/2*^*Mut*^ cases did not differ significantly with regard to sex, white blood cell count (WBC) or central nervous system (CNS) involvement (Table1). Despite an initial good treatment response (*IDH2*^*Mut*^ cases achieved 90% complete remission rate and *IDH2*^*Mut*^ did not confer increased poor prednisone response), patients with *IDH2*^Mut^ had an inferior outcome compared to *IDH2*^Wt^ (Table1, Fig. 1d, Additional file 2: Fig. S4), with an increased cumulative incidence of relapse (CIR) (4y-CIR: 78% vs 29%; specific hazard ratio (SHR) 4.3, 95%CI (2.0–9.2); *p* < 0.001) and a shorter overall survival (OS) (4y-OS: 30% vs 71%; hazard ratio: 3.6, 95%CI (1.7–7.7); *p* = 0.001). In multivariate analysis considering variables associated with CIR and OS in univariate analyses as covariates, *IDH2*^*Mut*^ predicted a trend for lower OS (HR: 1.98, 95%CI (0.86–4.57); *p* = 0.11) and statistically higher CIR (SHR, 4.06, 95%CI (1.84–8.96), *p* = 0.001) even after adjustment on the 4-gene *NOTCH1/FBXW7/RAS/PTEN (NFRP)* classifier which identified poor prognosis patients in both GRAALL and FRALLE trials [3, 4]. Conversely to *IDH2*^*Mut*^, *IDH1*^*Mut*^ was not associated with poor prognostic impact in T-ALL (4y-CIR: 25% vs 29%, *p* = 0.75 and 4y-OS: 80% vs 71%, *p* = 0.61).

We provide the largest comprehensive analysis of *IDH1* and *IDH2* mutations in T-ALL and highlight for the first time both their clinical profile and, most importantly, the extremely poor prognosis impact associated with *IDH2*^Mut^. We describe the specific oncogenetic landscape of *IDH1/2*^*Mut*^ and interestingly report that *IDH2*^Mut^ T-ALL conversely to *IDH1*^Mut^ were associated with an immature phenotype and alterations such as *RAS* mutations, transcription factors alterations (*ETV6*, *IKZF1*) and epigenetic regulators alterations (*TET2*, *DNMT3A*).

Recent studies have shed light on new prognostic factor in T-ALL allowing sharper prediction of the risk of relapse (e.g., *NFRP* classifier, level of MRD1, *IKZF1* alterations) [3, 4, 27]. Despite this, a significant number of T-ALL relapses remain unpredicted, so new predictive markers are needed, given the extremely poor prognosis associated with T-ALL relapse. We therefore consider that *IDH2*^Mut^ T-ALL cases should be identified at diagnosis to benefit from therapeutic intensification and/or specific *IDH2*^Mut^ inhibitors [15].

## Supplementary Information

**Additional file 1.** **Supplemental Table 1:** Custom capture Nextera XT gene panel. **Supplemental Table 2:** *IDH1* and *IDH2* mutations identified in 1085 patients with T-ALL. **Supplemental Table 3:** Chemotherapy in the FRALLE 2000 standard risk group T1 and high risk T2.

**Additional file 2.** **Figure S1:** Lollipop plots indicating the observed mutations for *IDH1* and *IDH2* in the present series confront with Cosmic-reported mutations for AML and AITL. **Figure S2:** Lollipop plots indicating the observed mutations for *IDH1* and *IDH2* affecting patients included in FRALLE and GRAALL protocol. **Figure S3:** Variant Allele Frequency (VAF) of individual *IDH1* and *IDH2* mutations observed in 1085 T-ALL. **Figure S4:** OS and CIR according to the *IDH1* or *IDH2*^Mut^ status in the two subgroups (FRALLE and GRALL 03/05). **Figure S5:** General design of FRALLE 2000 T guidelines.

## Data Availability

The datasets used and/or analyzed during the current study are available from the corresponding author on reasonable request.

## Abbreviations

*IDH1/2*^*Mut*^: *IDH1*-*IDH2* Mutations
AML: Acute myeloid leukemia
T-ALL: T-cell acute lymphoblastic leukemia
*NFRP*: *NOTCH1/FBXW7/RAS/PTEN*
CNS: Central nervous system
WBC: White blood cell count
NOS: Not otherwise specified
*T*_FH_: *T*-Follicular helper
ETP: Early thymic precursor
MRD: Minimal residual disease
