# Supplementary material for: Oncogenetic landscape and clinical impact of IDH1 and IDH2 mutations in T-ALL
Source: J Hematol Oncol. 2021 May 3;14:74. doi: 10.1186/s13045-021-01068-4 (PMC8091755; doi:10.1186/s13045-021-01068-4)
Supplement: Supplementary file 1 — Additional file 1. Supplemental Table 1: Custom capture Nextera XT gene panel. Supplemental Table 2: IDH1 and IDH2 mutations identified in 1085 patients with T-ALL. Supplemental Table 3: Chemotherapy in the FRALLE 2000 standard risk group T1 and high risk T2. [file 13045_2021_1068_MOESM1_ESM.docx]

***SUPPLEMENTAL DATA***

**Oncogenetic Landscape and Clinical Impact of *IDH1* and *IDH2* Mutations in T-ALL**

## **1-** SUPPLEMENTAL TABLES

- Supplemental **Table 1:** Custom capture Nextera XT gene panel

- Supplemental **Table 2**: *IDH1* and *IDH2* mutations identified in 1085 patients with T-ALL

- Supplemental **Table 3**: Chemotherapy in the FRALLE 2000 standard risk group T1 and high risk T2

## **2-** SUPPLEMENTAL FIGURES

- Supplemental **Figure 1**: Lollipop plots indicating the observed mutations for *IDH1* and *IDH2* in the present series confront with Cosmic-reported mutations for AML and AITL

- Supplemental **Figure 2**: Lollipop plots indicating the observed mutations for *IDH1* and *IDH2* affecting patients included in FRALLE and GRAALL protocol

- Supplemental **Figure 3**: Variant Allele Frequency (VAF) of individual *IDH1* and *IDH2* mutations observed in 1085 T-ALL

- Supplemental **Figure 4**: OS and CIR according to the *IDH1* or *IDH2*^Mut^ status in the two subgroups (FRALLE and GRALL 03/05)

- Supplemental **Figure 5**: General design of FRALLE 2000 T guidelines

## 1- SUPPLEMENTAL TABLES

**Supplemental Table 1.** Custom capture Nextera XT gene panel

**Supplemental Table 2.** *IDH1* and *IDH2* mutations identified in 1085 patients with T-ALL

**Supplemental Table 3.** Chemotherapy in the FRALLE 2000 standard risk group T1 and high risk T2

## 2- SUPPLEMENTAL FIGURES - LEGENDS

**Supplemental Figure 1.** Lollipop plots indicating the observed mutations for *IDH1* and *IDH2* in the present series confront with Cosmic-reported mutations for Acute Myeloid Leukemia (AML) and angio-immunoblastic T cell lymphoma (AITL).

**Supplemental Figure 2.** Lollipop plots indicating the observed mutations for *IDH1* and *IDH2* affecting patients included in FRALLE and GRAALL protocol

**Supplemental Figure 3.** Variant Allele Frequency (VAF) of individual *IDH1* and *IDH2* mutations observed in 1085 T-ALL

**Supplemental Figure 4.** OS and CIR according to the *IDH1* or *IDH2*^Mut^ status in the two subgroups (FRALLE and GRALL 03/05)

**Supplemental Figure 5.** General design of FRALLE 2000 T guidelines
