# Supplementary material for: Oncogenetic landscape and clinical impact of IDH1 and IDH2 mutations in T-ALL
Source: J Hematol Oncol. 2021 May 3;14:74. doi: 10.1186/s13045-021-01068-4 (PMC8091755; doi:10.1186/s13045-021-01068-4)
Supplement: Supplementary file 2 — Additional file 2. Figure S1: Lollipop plots indicating the observed mutations for IDH1 and IDH2 in the present series confront with Cosmic-reported mutations for AML and AITL. Figure S2: Lollipop plots indicating the observed mutations for IDH1 and IDH2 affecting patients included in FRALLE and GRAALL protocol. Figure S3: Variant Allele Frequency (VAF) of individual IDH1 and IDH2 mutations observed in 1085 T-ALL. Figure S4: OS and CIR according to the IDH1 or IDH2Mut status in the two subgroups (FRALLE and GRALL 03/05). Figure S5: General design of FRALLE 2000 T guidelines. [file 13045_2021_1068_MOESM2_ESM.pdf]

Supplemental Figures *IDH1 IDH2 Mutations* in T-ALL

S.Fig.1

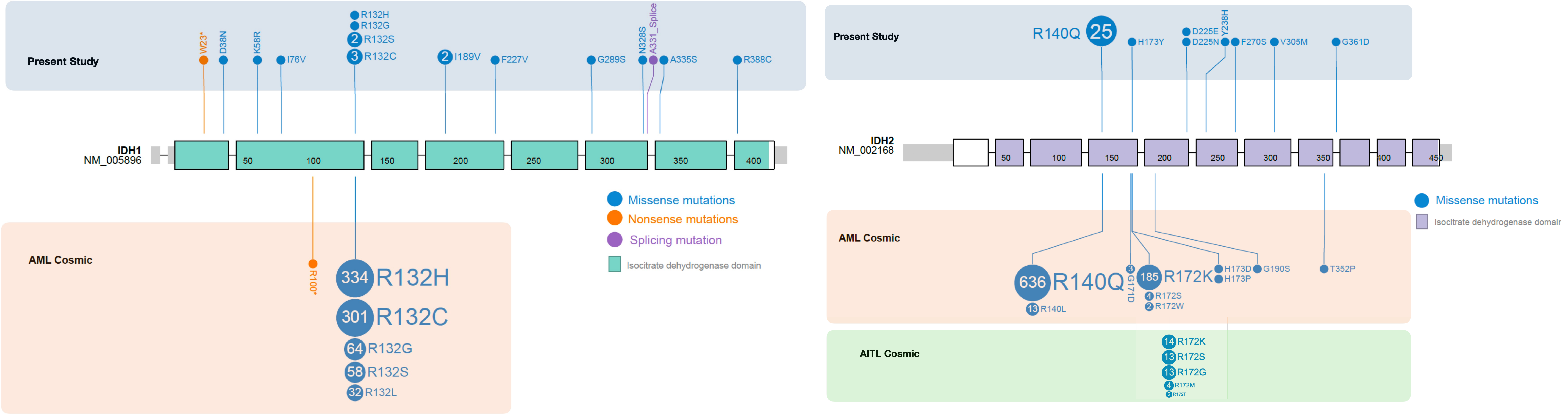

S.Fig.2

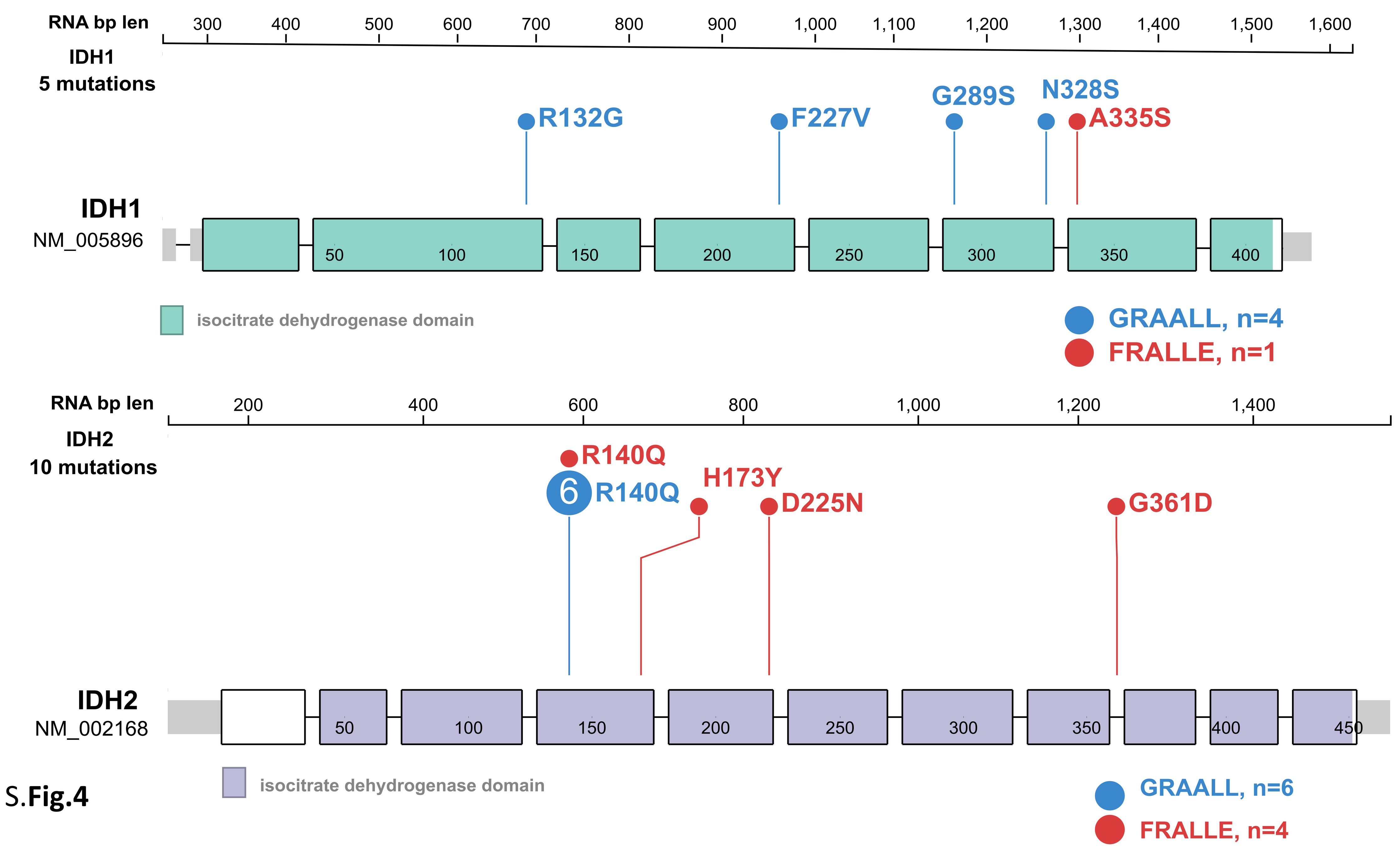

S.Fig.3

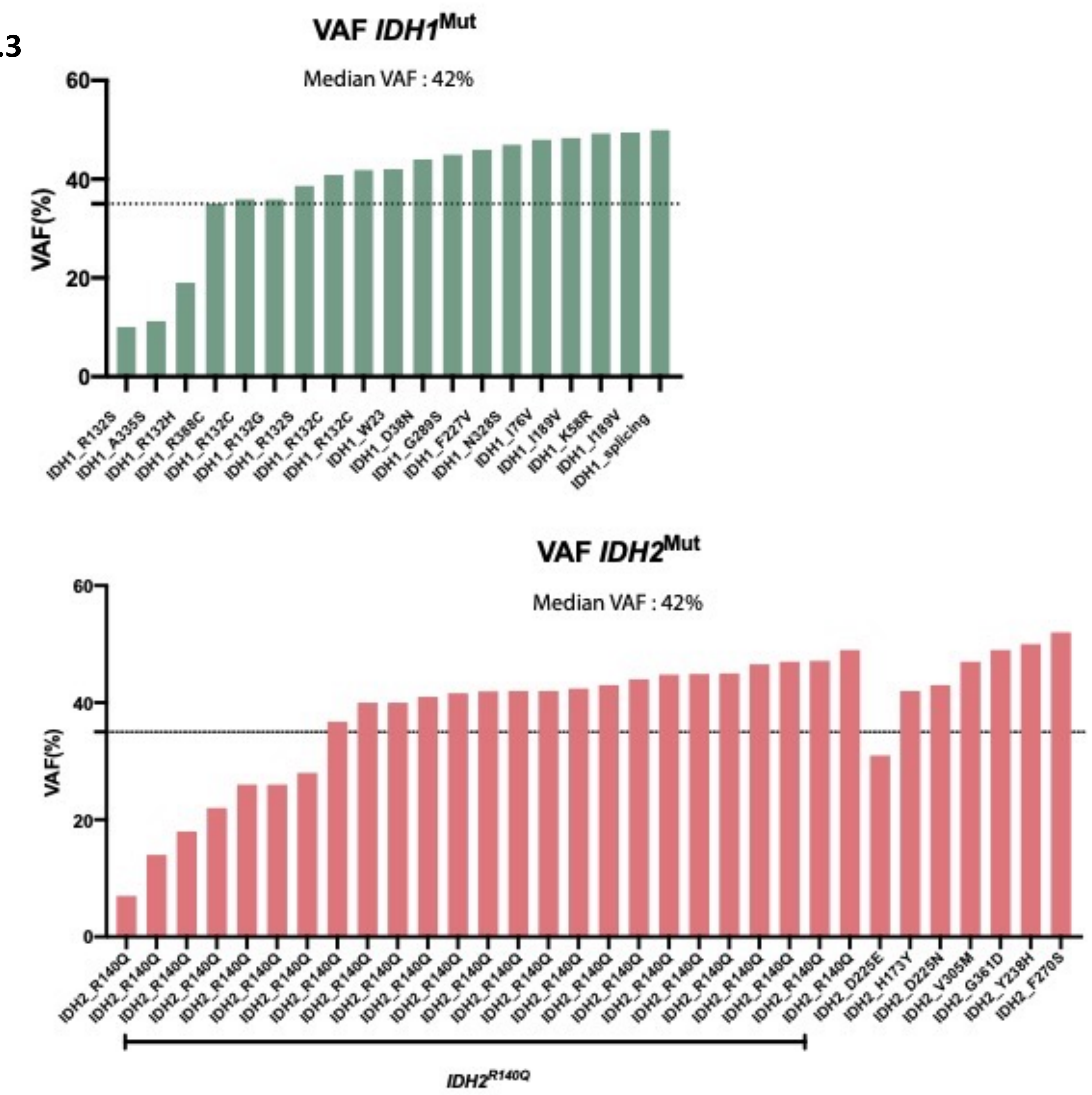

S.Fig.4

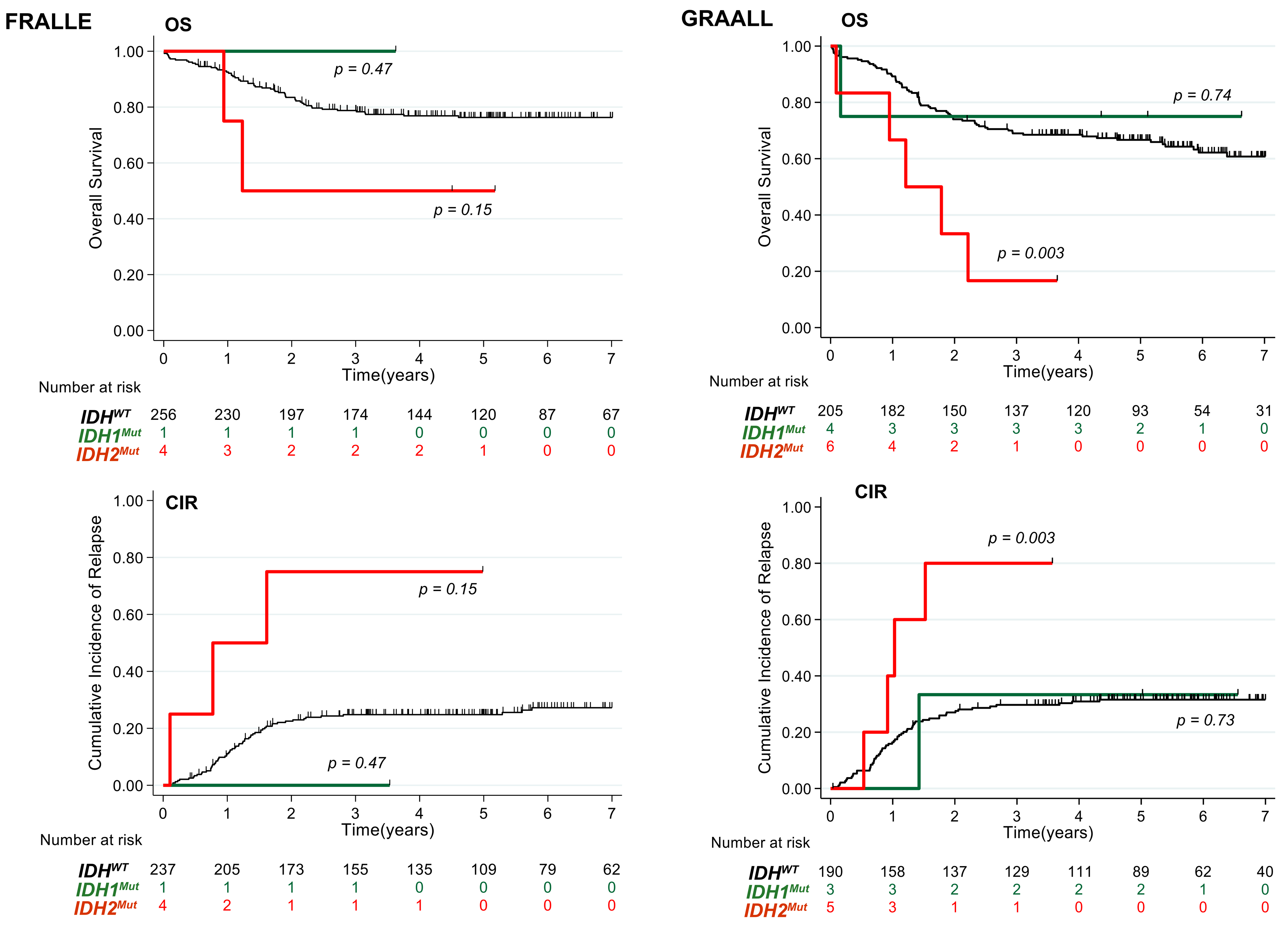

S.Fig.5

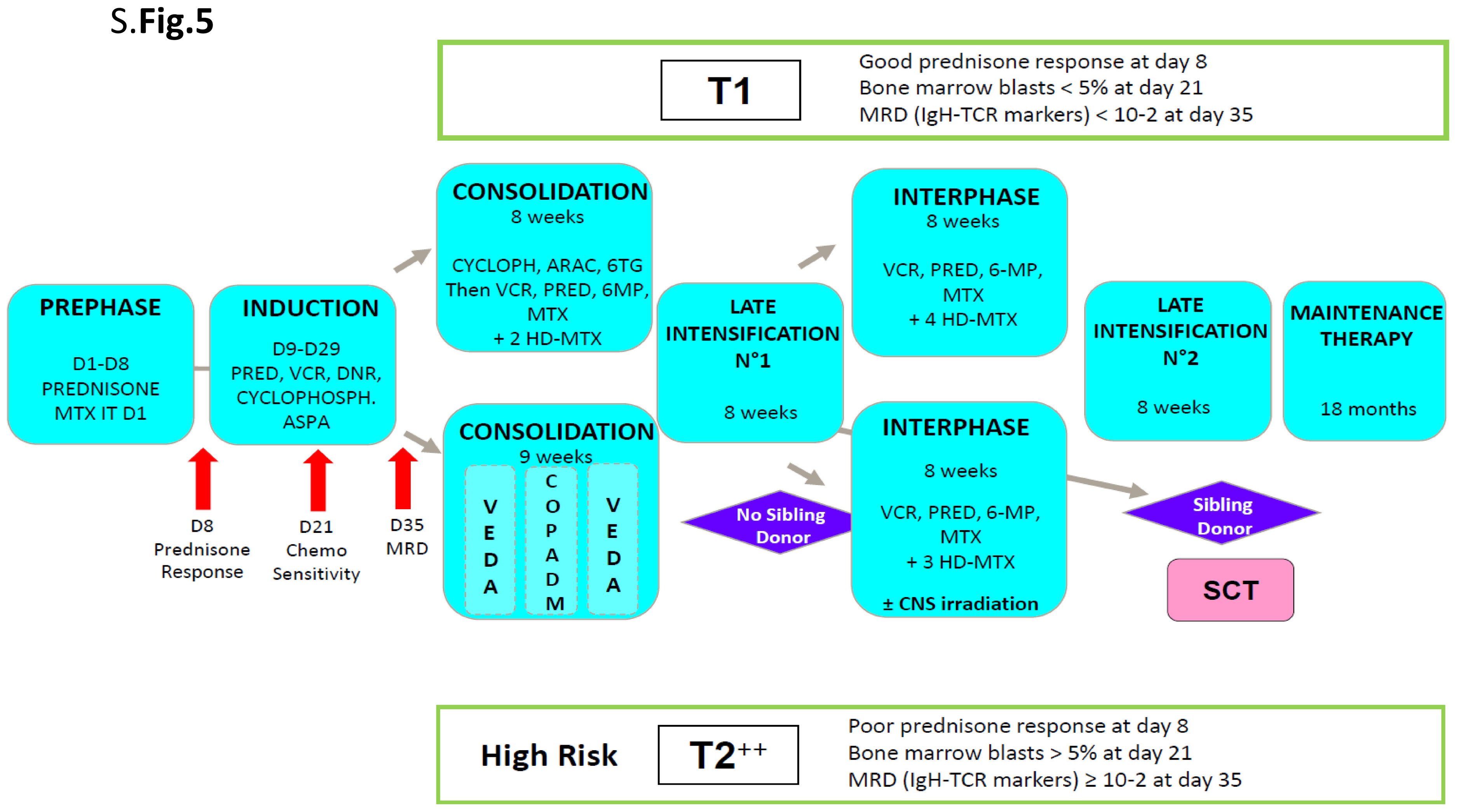

++ Patients treated according T2 group were eligible for SCT after late intensification n°1 when a sibling donor was available
